# Association of Preoperative Body Weight and Weight Loss With Risk of Death After Bariatric Surgery

**DOI:** 10.1001/jamanetworkopen.2020.4803

**Published:** 2020-05-14

**Authors:** Yangbo Sun, Buyun Liu, Jessica K. Smith, Marcelo L. G. Correia, Dana L. Jones, Zhanyong Zhu, Adeyinka Taiwo, Lisa L. Morselli, Katie Robinson, Alexander A. Hart, Linda G. Snetselaar, Wei Bao

**Affiliations:** 1 Department of Epidemiology, University of Iowa College of Public Health, Iowa City; 2 Carver College of Medicine, Department of Surgery, University of Iowa, Iowa City; 3 Carver College of Medicine, Department of Internal Medicine, University of Iowa, Iowa City; 4 Fraternal Order of Eagles Diabetes Research Center, University of Iowa, Iowa City; 5 Department of Plastic Surgery, Renmin Hospital of Wuhan University, Wuhan, China; 6 now with Scientific and Medical Affairs, Abbott Nutrition, Columbus, Ohio; 7 Obesity Research and Education Initiative, University of Iowa, Iowa City

## Abstract

**Importance:**

Perception of weight loss requirements before bariatric surgery varies among patients, physicians, and health insurance payers. Current clinical guidelines do not require preoperative weight loss because of a lack of scientific support regarding its benefits.

**Objective:**

To examine the association of preoperative body mass index (BMI) and weight loss with 30-day mortality after bariatric surgery.

**Design, Setting, and Participants:**

This cohort study used data from 480 075 patients who underwent bariatric surgery from 2015 to 2017 in the Metabolic and Bariatric Surgery Accreditation and Quality Improvement Program, which covers more than 90% of all bariatric surgery programs in the United States and Canada. Clinical and demographic data were collected at all participating institutions using a standardized protocol. Data analysis was performed from December 2018 to November 2019.

**Exposures:**

Preoperative BMI and weight loss.

**Main Outcomes and Measures:**

30-day mortality after bariatric surgery.

**Results:**

Of the 480 075 patients (mean [SD] age 45.1 [12.0] years; 383 265 [79.8%] women), 511 deaths (0.1%) occurred within 30 days of bariatric surgery. Compared with patients with a preoperative BMI of 35.0 to 39.9, the multivariable-adjusted odds ratios for 30-day mortality for patients with preoperative BMI of 40.0 to 44.9, 45.0 to 49.9, 50.0 to 54.9, and 55.0 and greater were 1.37 (95% CI, 1.02-1.83), 2.19 (95% CI, 1.64-2.92), 2.61 (95% CI, 1.90-3.58), and 5.03 (95% CI, 3.78-6.68), respectively (*P* for trend < .001). Moreover, compared with no preoperative weight loss, the multivariable-adjusted odds ratios for 30-day mortality for patients with weight loss of more than 0% to less than 5.0%, 5.0% to 9.9%, and 10.0% and greater were 0.76 (95% CI, 0.60-0.96), 0.69 (95% CI, 0.53-0.90), and 0.58 (95% CI, 0.41-0.82), respectively (*P* for trend = .003).

**Conclusions and Relevance:**

In this study, even moderate weight loss (ie, >0% to <5%) before bariatric surgery was associated with a lower risk of 30-day mortality. These findings may help inform future updates of clinical guidelines regarding bariatric surgery.

## Key Points

**Question:**

Are preoperative body mass index and weight loss associated with 30-day mortality after bariatric surgery?

**Findings:**

In a cohort study of 480 075 patients who underwent bariatric surgery from 2015 to 2017, even modest weight loss before bariatric surgery was associated with lower risk of 30-day mortality after the procedure. Compared with patients with no preoperative weight loss, patients with weight loss greater than 0% to less than 5.0%, 5.0% to 9.9%, and 10.0% and greater had 24%, 31%, and 42%, respectively, lower risk of 30-day mortality.

**Meaning:**

In this study of patients who underwent bariatric surgery, even moderate weight loss (ie, >0% to <5%) before the procedure was associated with lower risk of 30-day mortality; these findings may help to inform future updates of clinical guidelines regarding bariatric surgery.

## Teaser

This cohort study examines the association of preoperative body mass index and weight loss with 30-day mortality after bariatric surgery.

## Introduction

Obesity is a rising epidemic in the US and worldwide. Obesity is associated with increased all-cause mortality, and more than 300 000 deaths annually in the US are attributed to obesity.^1,2^ In addition to the health consequences of obesity, the direct medical costs related to adult obesity in the US has been estimated at more than $315 billion yearly.^3^ Bariatric surgery has been shown to be the most effective and durable treatment for clinically morbid obesity (ie, body mass index [BMI; calculated as weight in kilograms divided by height in meters squared] ≥35.0 with comorbidities), which is difficult to reverse through traditional approaches such as lifestyle intervention.^4^ There has been long-standing uncertainty and debate regarding the value of preoperative weight loss as a requirement for bariatric surgery. Perception of preoperative weight loss requirements varies among patients, physicians, hospitals, and health insurance payers. Current clinical guidelines by the American Society for Metabolic and Bariatric Surgery do not recommend preoperative weight loss because of a lack of scientific support regarding its benefits.^5^ Clearly, there is an urgent and critical need to clarify the potential influence of preoperative weight loss on postoperative outcomes after bariatric surgery.

One of the major indicators for surgical outcomes is 30-day mortality after the procedure. While it is important to aim for reducing the death rate to 0 for any surgical procedure, this is especially important for bariatric surgery because most bariatric operations are voluntary and patients are not expected to die in the short term if the surgery is not performed. Several previous studies have examined the association of preoperative weight loss with postoperative weight loss, and they have yielded inconsistent findings.^6,7,8^ So far, the association of preoperative weight loss with 30-day mortality after bariatric surgery remains unclear.^9^ Previous studies pertaining to bariatric surgery outcomes usually have limited sample sizes or limited procedure types, which has compromised their ability to detect an association with a rare outcome, such as death.^9,10^ Therefore, we aimed to examine the associations of preoperative BMI and weight loss with 30-day mortality following bariatric surgery using a large database of approximately 500 000 patients who underwent bariatric surgery in the United States and Canada.

## Methods

### Study Population

We used data from the Metabolic and Bariatric Surgery Accreditation and Quality Improvement Program (MBSAQIP) Participant Use Data File (PUF) from 2015 to 2017.^11^ The PUF includes data that were collected prospectively from 832 participating centers between January 1, 2015, and December 31, 2017, in the United States and Canada. These data are abstracted from medical records by certified metabolic and bariatric surgical clinical reviewers who undergo database-specific trainings and must pass an annual certification exam. Preoperative and demographic variables, comorbidities, information on the procedure, and 30-day outcomes are collected. Data integrity is ensured by an audit process requiring a disagreement rate of less than 5%. The MBSAQIP covers more than 90% of all bariatric surgery programs in the United States and Canada.^11^ The PUF from 2015 to 2017 includes 555 239 cases performed in all MBSAQIP-participating institutions during that period.^12,13,14,15^ The University of Iowa institutional review board determined that this analysis was exempt from review and informed consent given the use of deidentified data. The present study was performed and reported in accordance with the Strengthening the Reporting of Observational Studies in Epidemiology (STROBE) reporting guideline for cohort studies.

For this analysis, we initially identified 505 287 patients aged 18 years or older with complete information on preoperative weight measurement and 30-day vital status. We excluded 22 277 patients (4.4%) whose highest recorded preoperative BMI was less than 35.0 and 2935 participants (0.6%) with weight gain before surgery, resulting in 480 075 patients (95.0%) in our analysis.

### Exposure Assessment

The highest recorded preoperative weight of the patient was measured at the bariatric center within 1 year before the principal operation. The preoperative weight closest to the day of surgery was defined as the most recent weight documented in the medical record within 30 days before the principal operation or at the time the patient was being considered a candidate for surgery. Height was the patient’s most recent height documented in the medical record within 30 days before the principal operation or at the time the patient was being considered a candidate for surgery. Weight loss (in pounds) was defined as the difference between the highest recorded preoperative weight and preoperative weight recorded closest to the procedure. The BMI reduction was the difference between the highest recorded preoperative BMI and preoperative BMI closest to the day of the operation.

### Outcome Assessment

The outcome was death within 30 days of bariatric surgery. This information was retrieved from the reporting of mortality up to and including the 30th day after the operation in all MBSAQIP-participating institutions.

### Covariate Assessments

Demographic details were collected for all patients, including age, sex, and race/ethnicity. Patient race/ethnicity was categorized as non-Hispanic white, non-Hispanic black, Hispanic, and other. Patients were categorized as currently smoking tobacco or not currently smoking tobacco based on information about smoking cigarettes at any point within the 12 months before admission for surgery. Patient comorbidities included diabetes; hypertension; hyperlipidemia; obstructive sleep apnea; chronic obstructive pulmonary disease; previous percutaneous coronary intervention or percutaneous transluminal coronary angioplasty; previous major cardiac surgery; renal insufficiency, receiving dialysis, or requiring dialysis 2 weeks before the principal operation; deep vein thrombosis requiring therapy; presence of inferior vena cava filter; and therapeutic anticoagulation. The number of comorbidities was further categorized as 0, 1, or 2 or more.

### Statistical Analysis

Comparisons of covariates among different groups were performed using analysis of variance for continuous variables and χ^2^ tests for categorical variables. Dummy categories were created for missing values. We used multivariable logistic regression to estimate odds ratios (ORs) and 95% CIs for 30-day mortality associated with preoperative BMI and preoperative weight loss or BMI reduction. We fit 3 different adjusted models. Model 1 was adjusted for age, sex, and race/ethnicity. Model 2 was adjusted for covariates included in model 1 plus *Current Procedural Terminology* principal operation, history of bariatric surgery, whether the procedure was a revision or conversion, and whether the patient underwent emergency surgery during the hospital admission. Model 3 was adjusted for covariates included in model 2 plus preoperative smoking status and comorbidities. For the analysis of preoperative weight loss or BMI reduction with mortality risk, the highest recorded preoperative BMI was also adjusted in the model. Tests for linear trends were conducted across categories of preoperative BMI or weight loss by assigning the median value for each category and fitting this continuous variable in the models.

We performed sensitivity analyses by excluding patients who had previous bariatric surgery, revision bariatric surgery, or emergency surgery and restricting the analysis to 2 main bariatric types (ie, Roux-en-Y gastric bypass and sleeve). Data analysis was performed from December 2018 to November 2019, using survey procedures in SAS statistical software version 9.4 (SAS Institute). *P* < .05 was considered statistically significant, and all tests were 2-tailed.

## Results

We included 480 075 patients who received bariatric surgery with a mean (SD) age of 45.1 (12.0) years (range, 18-80 years), including 383 265 (79.8%) women. A total of 511 (0.1%) deaths occurred during operation or within 30 days following bariatric surgery. Mean percentage of reduction in body weight before surgery was 4.0% (SD, 4.4%; range, 0%-70.2%). Compared with patients without weight loss before surgery, patients with greater weight loss (ie, ≥10%) before bariatric surgery were slightly older (mean [SD] age, 44.7 [11.9] years vs 47.1 [11.9] years; *P* < .001) and more likely to be male (15 519 [18.0%] vs 8984 [25.4%]; *P* < .001) and non-Hispanic white (46 995 [54.6%] vs 25 017 [70.6%]; *P* < .001) (Table 1). Compared with patients without weight loss before surgery, patients with greater weight loss were more likely to undergo Roux-en-Y gastric bypass (18 951 [22.0%] vs 10 665 [30.1%]; *P* < .001). Compared with patients without weight loss before surgery, those with greater weight loss had higher highest recorded preoperative BMI (mean [SD], 45.9 [7.9] vs 51.0 [10.3]; *P* < .001) and lower preoperative BMI closest to the day of surgery (mean [SD], 45.9 [7.9] vs 43.4 [8.7]; *P* < .001), and they were less likely to have smoked tobacco during the past year (8006 [9.3%] vs 2358 [6.7%]; *P* < .001) and more likely to have 2 or more comorbid conditions at baseline (30 558 [35.5%] vs 17 541 [49.5%]; *P* < .001).

**Table 1.** Demographic Characteristics According to Weight Loss Percentage Before Bariatric Surgery

Characteristic	No. (%)					*P* value
	Whole sample (N = 480 075)	Weight loss, %				
		0 (n = 86 063)	>0 to <5.0 (n = 240 242)	5.0-9.9 (n = 118 142)	≥10.0 (n = 35 446)	
Age, mean (SD), y	45.1 (12.0)	44.7 (11.9)	44.7 (11.9)	46.0 (12.0)	47.1 (11.9)	<.001
Men	96 810 (20.2)	15 519 (18.0)	44 819 (18.6)	27 488 (23.3)	8984 (25.4)	<.001
Race/ethnicity						
Non-Hispanic white	292 294 (60.9)	46 995 (54.6)	141 845 (59.0)	78 437 (66.4)	25 017 (70.6)	<.001
Non-Hispanic black	77 191 (16.1)	15 465 (18.0)	41 852 (17.4)	16 256 (13.8)	3618 (10.2)	
Hispanic	55 985 (11.7)	11 063 (12.9)	29 627 (12.3)	11 867 (10.0)	3428 (9.7)	
Other	11 660 (2.4)	2052 (2.4)	6148 (2.6)	2729 (2.3)	731 (2.1)	
Missing	42 945 (9.0)	10 488 (12.2)	20 952 (8.7)	8853 (7.5)	2652 (7.5)	
*CPT* principal operation						
Sleeve	305 332 (63.6)	52 798 (61.4)	155 620 (64.8)	76 782 (65.0)	20 132 (56.8)	<.001
Laparoscopic or open RYGB	126 362 (26.3)	18 951 (22.0)	62 388 (26.0)	34 358 (29.1)	10 665 (30.1)	
Band	25 831 (5.4)	9152 (10.6)	12 579 (5.2)	3109 (2.6)	991 (2.8)	
Other	22 550 (4.7)	5162 (6.0)	9837 (4.1)	3893 (3.3)	3658 (10.3)	
Emergency surgery	1908 (0.4)	778 (0.9)	446 (0.2)	205 (0.2)	479 (1.4)	<.001
History of bariatric surgery	56 659 (11.8)	16 098 (18.7)	27 679 (11.5)	8142 (6.9)	4740 (13.4)	<.001
Revisional or conversional procedure	54 211 (11.3)	15 924 (18.5)	26 354 (11.0)	7442 (6.3)	4491 (12.7)	<.001
Smoked tobacco within 1 y	40 980 (8.5)	8006 (9.3)	24 550 (9.0)	118 142 (7.7)	2358 (6.7)	<.001
Comorbid conditions, No.						
0	147 255 (30.7)	31 100 (36.1)	76 931 (32.0)	3.689 (26.0)	8535 (24.1)	<.001
1	132 888 (27.7)	24 375 (28.3)	67 296 (28.0)	31 847 (27.0)	9370 (26.4)	
≥2	199 932 (41.7)	30 588 (35.5)	96 197 (40.0)	55 606 (47.1)	17 541 (49.5)	
Diabetes	121 942 (25.4)	18 518 (21.5)	59 846 (24.9)	33 565 (28.4)	10 013 (28.3)	<.001
Hypertension	231 489 (48.2)	38 323 (44.5)	112 905 (47.0)	61 448 (52.0)	18 813 (53.1)	<.001
Hyperlipidemia	114 432 (23.8)	17 817 (20.7)	55 432 (23.1)	31 487 (26.7)	9696 (27.4)	<.001
Obstructive sleep apnea	177 693 (37.0)	25 837 (30.0)	84 736 (35.2)	50 470 (42.7)	16 650 (47.0)	<.001
COPD	8375 (1.7)	1327 (1.5)	3977 (1.7)	2226 (1.9)	845 (2.4)	<.001
Percutaneous coronary intervention	9941 (2.1)	1594 (1.9)	4776 (2.0)	2733 (2.3)	838 (2.4)	<.001
Previous cardiac surgery	5409 (1.1)	897 (1.0)	2594 (1.1)	1485 (1.3)	433 (1.2)	<.001
Renal insufficiency, requiring dialysis, or receiving dialysis	3682 (0.8)	660 (0.8)	1732 (0.7)	957 (0.8)	333 (0.9)	<.001
Vein thrombosis requiring therapy	8172 (1.7)	1260 (1.5)	3861 (1.6)	2194 (1.9)	857 (2.4)	<.001
Inferior vena cava filter	3781 (0.8)	501 (0.6)	1842 (0.8)	1065 (0.9)	373 (1.1)	<.001
Therapeutic anticoagulation	13 079 (2.7)	1972 (2.3)	6086 (2.5)	3636 (3.1)	1385 (3.9)	<.001
Highest recorded preoperative BMI, mean (SD)	47.1 (8.2)	45.9 (7.9)	46.5 (7.7)	48.1 (8.3)	51.0 (10.3)	<.001
Preoperative BMI closest to the day of surgery, mean (SD)	45.2 (7.8)	45.9 (7.9)	45.3 (7.5)	44.7 (7.7)	43.4 (8.7)	<.001

Abbreviations: BMI, body mass index (calculated as weight in kilograms divided by height in meters squared); COPD, chronic obstructive pulmonary disease; *CPT*, *Current Procedural Terminology*; RYGB, Roux-en-Y gastric bypass.

Greater preoperative BMI was associated with higher risk of 30-day mortality after adjusting for age, sex, and race/ethnicity (eg, BMI ≥55.0: OR, 5.29; 95% CI, 4.01-6.99) (Table 2). After adjustment for a variety of potential confounders in model 3, compared with patients with preoperative BMI closest to the day of surgery between 35.0 and 39.9, the multivariable-adjusted ORs for 30-day mortality for patients with preoperative BMI closest to day of surgery of 40.0 to 44.9, 45.0 to 49.9, 50.0 to 54.9, and 55.0 or greater were 1.37 (95% CI, 1.02-1.83), 2.19 (95% CI, 1.64-2.92), 2.61 (95% CI, 1.90-3.58), and 5.03 (95% CI, 3.78-6.68), respectively (*P* for trend < .001) (Table 2). Similarly, compared with patients with highest recorded preoperative BMI of 35.0 to 39.9, the multivariable-adjusted ORs for 30-day mortality for patients with highest recorded preoperative BMI of 40.0 to 44.9, 45.0 to 49.9, 50.0 to 54.9, and 55.0 or greater were 1.78 (95% CI, 1.24-2.54), 2.26 (95% CI, 1.58-3.24), 3.22 (95% CI, 2.23-4.65), and 5.21 (95% CI, 3.69-7.35), respectively (*P* for trend < .001) (Table 3).

**Table 2.** Association of Preoperative BMI Closest to Bariatric Surgery With Intraoperative or 30-Day Postoperative Mortality

Model	OR (95% CI) by preoperative BMI					*P* for trend
	35.0-39.9 (n = 129 888)	40.0-44.9 (n = 146 559)	45.0-49.9 (n = 97 126)	50.0-54.9 (n = 55 155)	≥55.0 (n = 51 347)	
Deaths, No. (%)	84 (<0.1)	104 (<0.1)	112 (0.1)	76 (0.1)	135 (0.2)	NA
Model 1^a^	1 [Reference]	1.26 (0.95-1.70)	2.09 (1.57-2.77)	2.60 (1.90-3.56)	5.29 (4.01-6.99)	<.001
Model 2^b^	1 [Reference]	1.38 (1.03-1.84)	2.27 (1.70-3.02)	2.77 (2.02-3.81)	5.54 (4.18-7.35)	<.001
Model 3^c^	1 [Reference]	1.37 (1.02-1.83)	2.19 (1.64-2.92)	2.61 (1.90-3.58)	5.03 (3.78-6.68)	<.001

Abbreviations: BMI, body mass index (calculated as weight in kilograms divided by height in meters squared); NA, not applicable; OR, odds ratio.

^a^ Model 1 was adjusted for age, sex, and race/ethnicity.

^b^ Model 2 was adjusted for covariates included in model 1 plus* Current Procedural Terminology *principal operation, history of bariatric surgery, whether the bariatric surgery was a revision or conversion, and whether the patient underwent emergency surgery during the hospital admission.

^c^ Model 3 was adjusted for covariates included in model 2 plus smoking status and comorbidities (ie, 0, 1, or ≥2 conditions).

**Table 3.** Association of Highest Recorded Preoperative BMI With Intraoperative or 30-Day Postoperative Mortality

Model	OR (95% CI) by highest recorded preoperative BMI					*P* for trend
	35.0-39.9 (n = 82 634)	40.0-44.9 (n = 147 142)	45.0-49.9 (n = 109 179)	50.0-54.9 (n = 68 076)	≥55.0 (n = 73 044)	
Deaths, No. (%)	43 (<0.1)	107 (<0.1)	103 (<0.1)	94 (0.1)	164 (0.2)	NA
Model 1^a^	1 [Reference]	1.63 (1.15-2.33)	2.13 (1.49-3.04)	3.19 (2.22-4.59)	5.46 (3.89-7.68)	<.001
Model 2^b^	1 [Reference]	1.77 (1.24-2.53)	2.31 (1.61-3.31)	3.39 (2.35-4.89)	5.68 (4.03-8.02)	<.001
Model 3^c^	1 [Reference]	1.78 (1.24-2.54)	2.26 (1.58-3.24)	3.22 (2.24-4.65)	5.21 (3.69-7.36)	<.001

Abbreviations: BMI, body mass index (calculated as weight in kilograms divided by height in meters squared); NA, not applicable; OR, odds ratio.

^a^ Model 1 was adjusted for age, sex, and race/ethnicity.

^b^ Model 2 was adjusted for covariates included in model 1 plus* Current Procedural Terminology *principal operation, history of bariatric surgery, whether the bariatric surgery was a revision or conversion, and whether the patient underwent emergency surgery during the hospital admission.

^c^ Model 3 was adjusted for covariates included in model 2 plus smoking status and comorbidities (ie, 0, 1, or ≥2 conditions).

Greater weight loss before bariatric surgery was associated with lower risk of 30-day mortality. After adjustment for a variety of potential confounders in model 3, compared with patients with no preoperative weight loss, the multivariable-adjusted ORs for 30-day mortality for patients with percentage body weight reduction of more than 0% to less than 5.0%, 5.0% to 9.9%, and 10.0% or greater were 0.76 (95% CI, 0.60-0.96), 0.69 (95% CI, 0.53-0.90), and 0.58 (95% CI, 0.41-0.82), respectively (*P* for trend = .003) (Table 4). Similarly, compared with patients with no reduction in preoperative BMI, the multivariable-adjusted ORs for 30-day mortality for patients with BMI reduction of more than 0% to less than 5.0%, 5.0% to 9.9%, and 10.0% or greater were 0.75 (95% CI, 0.59-0.94), 0.69 (95% CI, 0.53-0.90), and 0.57 (95% CI, 0.40-0.81), respectively (*P* for trend = .003) (Table 5). The results were similar in the sensitivity analyses when patients who had previous bariatric surgery, revision bariatric surgery, or emergency surgery were excluded or when the analysis was restricted to patients who underwent Roux-en-Y gastric bypass or sleeve procedures only (eTable in the Supplement).

**Table 4.** Association of Weight Loss Percentage With Intraoperative or 30-Day Postoperative Mortality

Model	OR (95% CI) by weight loss percentage				*P* for trend
	0% (n = 86 063)	>0% to <5.0% (n = 240 424)	5.0%-9.9% (n = 118 142)	≥10.0% (n = 35 446)	
Deaths, No. (%)	105 (0.1)	230 (<0.1)	129 (0.1)	47 (0.1)	NA
Model 1^a^	1 [Reference]	0.73 (0.58-0.93)	0.67 (0.52-0.87)	0.65 (0.46-0.93)	.02
Model 2^b^	1 [Reference]	0.77 (0.61-0.98)	0.71 (0.54-0.93)	0.58 (0.41-0.82)	.003
Model 3^c^	1 [Reference]	0.76 (0.60-0.96)	0.69 (0.53-0.90)	0.58 (0.41-0.82)	.003

Abbreviations: NA, not applicable; OR, odds ratio.

^a^ Model 1 was adjusted for age, sex, race/ethnicity, and highest recorded preoperative body mass index.

^b^ Model 2 was adjusted for covariates included in model 1 plus* Current Procedural Terminology *principal operation, history of bariatric surgery, whether the bariatric surgery was a revision or conversion, and whether the patient underwent emergency surgery during the hospital admission.

^c^ Model 3 was adjusted for covariates included in model 2 plus smoking status and comorbidities (ie, 0, 1, or ≥2 conditions).

**Table 5.** Association of Percentage BMI Reduction With Intraoperative or 30-Day Postoperative Mortality

Model	OR (95% CI) by percentage BMI reduction				*P* for trend
	0% (n = 86 229)	>0% to <5.0% (n = 240 362)	5.0%-9.9% (n = 118 094)	≥10.0% (n = 35 390)	
Deaths, No. (%)	106 (0.1)	229 (<0.1)	129 (0.1)	47 (0.1)	NA
Model 1^a^	1 [Reference]	0.73 (0.58-0.91)	0.67 (0.52-0.87)	0.65 (0.46-0.92)	.02
Model 2^b^	1 [Reference]	0.76 (0.60-0.96)	0.70 (0.54-0.92)	0.58 (0.41-0.82)	.003
Model 3^c^	1 [Reference]	0.75 (0.59-0.95)	0.69 (0.53-0.90)	0.57 (0.40-0.81)	.003

Abbreviations: BMI, body mass index (calculated as weight in kilograms divided by height in meters squared); NA, not applicable; OR, odds ratio.

^a^ Model 1 was adjusted for age, sex, race/ethnicity, and highest recorded preoperative BMI.

^b^ Model 2 was adjusted for covariates included in model 1 plus* Current Procedural Terminology *principal operation, history of bariatric surgery, whether the bariatric surgery was a revision or conversion, and whether the patient underwent emergency surgery during the hospital admission.

^c^ Model 3 was adjusted for covariates included in model 2 plus smoking status and comorbidities (ie, 0, 1, or ≥2 conditions).

## Discussion

In this large binational cohort study of patients who underwent bariatric procedures, we found that higher preoperative BMI was associated with a higher risk of 30-day mortality following bariatric surgery after adjusting for a variety of covariates. Furthermore, weight loss, even at a moderate degree (ie, >0% to <5% weight loss), was associated with significantly lower risk of 30-day mortality following bariatric surgery.

To our knowledge, this is the largest data set analyzed to investigate the dose-response association of preoperative weight and weight loss with risk of 30-day mortality. Previous studies have examined the association of preoperative weight loss with several surgical outcomes, such as intraoperative and postoperative complications, operating time, and length of hospital stay.^6,7,16,17^ The association of preoperative weight loss with 30-day mortality was investigated in several studies; however, the findings have been inconclusive.^9^ A 2019 meta-analysis^9^ identified 4 randomized clinical trials and 12 cohort studies, but the pooled sample size (6060 patients) was insufficient to have a robust estimate for the association of preoperative weight loss with risk of mortality because the mortality event rate was low.^9^ Another observational study^10^ did not find a significant association of preoperative weight loss as a binary category (ie, yes or no) with 30-day mortality, probably because of restricted surgery types and thus restricted sample size.^10^

There are several potential explanations for our results. Previous studies have confirmed the beneficial association of weight loss with comorbid medical conditions, including predisposition to thromboembolism.^18,19^ Venous thromboembolism, including deep vein thrombosis and pulmonary embolism, is a leading cause of postoperative mortality in patients undergoing bariatric surgery^20^; thus, improvements in blood coagulopathy and fibrinolysis and an increase in antithrombin III associated with weight loss may be important factors in reducing postoperative mortality.^21^ Furthermore, modest weight loss has been shown to significantly reduce apneic episodes and improve gas exchange,^22,23^ which may in turn reduce the risk of perioperative death.^24^ Another possibility is that morbidity and mortality are associated with greater technical difficulty and complexity of surgery in patients with obesity.^25,26,27^ Preoperative weight loss leads to a reduced liver volume and thus a reduction in technical complexity owing to better visualization of target abdominal structures during the procedure.^6^ Preoperative weight loss under the guidance of the bariatric team and bariatric dietitian also improves nutrient status, which contributes to improved outcomes and reduced mortality.^28^ Finally, hyperglycemia has been shown to increase in-hospital mortality,^29^ which can be attenuated by better glycemic control as a result of weight loss.^30^

Our study has important clinical implications. Each year, hundreds of patients in the US die after undergoing bariatric surgery. Using a binational data set, our findings showed that even a small reduction in preoperative weight (ie, >0% to <5%) was associated with a 24% reduction in the odds of 30-day mortality following bariatric surgery. Although current clinical guidelines do not require preoperative weight loss and a decision to perform bariatric surgery should not be based on whether and how much preoperative weight loss is achieved, it may be beneficial for patients with obesity to be referred to an established weight loss program before surgery to reduce the risk of mortality.

### Strengths and Limitations

The strengths of our study include a large sample size, a diverse population, and rich information that allowed adjustment for potential confounders. Nevertheless, our study has several limitations. First, despite all the advantages of MBSAQIP, our study could not establish causality between preoperative weight loss and postoperative mortality because of the observational nature of the study design. However, because the mortality rate among patients who underwent bariatric surgery is low, it is less feasible to conduct a randomized clinical trial with a sufficiently large sample size to evaluate the effects of weight loss on mortality risk. Second, institution-specific variables (such as type of intervention used to promote preoperative weight loss and implementation of enhanced recovery after surgery protocols) and surgeon-specific variables (such as experience and surgical technique) may affect mortality outcomes, but such information is not available in the MBSAQIP to protect the privacy of participating institutions, practitioners, and patients. Third, information about whether the weight loss program was voluntary, mandated by insurance, or unintentional was not available; thus, it was impossible to evaluate if the associations would differ by programs or reasons for weight loss. Fourth, our results were limited to short-term mortality after bariatric surgery. Long-term risk of mortality associated with preoperative weight loss warrants further investigation.

## Conclusions

Our study showed that preoperative weight loss, even at a moderate degree (ie, >0% to <5%), was associated with lower risk of 30-day mortality following bariatric surgery. Further investigation is needed to replicate our findings in a setting with further information on how weight loss was achieved and to inform future updates of clinical guidelines regarding bariatric surgery.

## Notes

Supplement. **eTable.** Adjusted Odds Ratios for Preoperative Weight Loss Percentage and Intraoperative or 30-Day Postoperative Mortality in Sensitivity Analyses
