# Supplementary material for: Association of Preoperative Body Weight and Weight Loss With Risk of Death After Bariatric Surgery
Source: JAMA Netw Open. 2020 May 14;3(5):e204803. doi: 10.1001/jamanetworkopen.2020.4803 (PMC7225906; doi:10.1001/jamanetworkopen.2020.4803)
Supplement: Supplement. — eTable. Adjusted Odds Ratios for Preoperative Weight Loss Percentage and Intraoperative or 30-Day Postoperative Mortality in Sensitivity Analyses [file jamanetwopen-3-e204803-s001.pdf]

Supplementary Online Content

Sun Y, Liu B, Smith JK, et al. Association of preoperative body weight and weight loss with risk of death after bariatric surgery. *JAMA Netw Open*. 2020;3(5):e204803. doi:10.1001/jamanetworkopen.2020.4803

eTable. Adjusted Odds Ratios for Preoperative Weight Loss Percentage and Intraoperative or 30-Day Postoperative Mortality in Sensitivity Analyses

This supplementary material has been provided by the authors to give readers additional information about their work.

© 2020 Sun Y et al. *JAMA Network Open.*

eTable. Adjusted Odds Ratios for Preoperative Weight Loss Percentage and Intraoperative or 30-Day Postoperative Mortality in Sensitivity Analyses

	[a]Weight loss %				
	0	<5	5-9.9	≥10.0	*P* for trend
No. of cases/patients	77/70139	193/214070	117/110700	31/30955	
Model 1	Reference	0.77(0.59, 1.01)	0.72(0.54, 0.96)	0.52(0.34, 0.80)	0.004
Model 2	Reference	0.75(0.58, 0.98)	0.70(0.52, 0.94)	0.49(0.32, 0.75)	0.002
Model 3	Reference	0.74(0.57, 0.97)	0.69(0.51, 0.92)	0.49(0.32, 0.75)	0.002
	[b]BMI reduction %				
	0	<5	5-9.9	≥10.0	*P* for trend
No. of cases/patients	77/70261	193/214061	117/110639	31/30903	
Model 1	Reference	0.77(0.59, 1.01)	0.72(0.54, 0.96)	0.53(0.34, 0.80)	0.005
Model 2	Reference	0.76(0.58, 0.99)	0.70(0.53, 0.94)	0.50(0.32, 0.76)	0.002
Model 3	Reference	0.74(0.57, 0.97)	0.69(0.52, 0.92)	0.49(0.32, 0.75)	0.002
	[c]Weight loss %				
	0	<5	5-9.9	≥10.0	*P* for trend
No. of cases/patients	47/52798	104/155620	56/76782	12/20132	
Model 1	Reference	0.70(0.50, 0.99)	0.61(0.41, 0.90)	0.38(0.20, 0.73)	0.002
Model 2[d]	Reference	0.71(0.50, 1.01)	0.62(0.42, 0.92)	0.39(0.21, 0.74)	0.003
Model 3	Reference	0.70(0.49, 0.99)	0.61(0.41, 0.90)	0.39(0.20, 0.73)	0.003
	[e]Weight loss %				
	0	<5	5-9.9	≥10.0	*P* for trend
No. of cases/patients	40/18993	89/62375	57/34342	18/10652	
Model 1	Reference	0.63(0.43, 0.91)	0.61(0.41, 0.92)	0.51(0.29, 0.90)	0.04
Model 2[f]	Reference	0.64(0.44, 0.93)	0.63(0.42, 0.95)	0.52(0.29, 0.91)	0.06
Model 3	Reference	0.63(0.43, 0.91)	0.62(0.41, 0.93)	0.51(0.29, 0.90)	0.05

Model 1: Adjusted for age, sex, race/ethnicity, and highest recorded pre-operative BMI.
Model 2: Adjusted for covariates included in model 1 plus CPT principal operative procedure, and if it was a revisional or conversional procedure.
Model 3: Adjusted for covariates included in model 2 plus smoking status and comorbidities (0, 1 or ≥2 conditions).
[a]The association between pre-operative weight loss percentage and intra-operative or 30-day post-operative mortality among 425864 participants excluding patients who had previous bariatric surgery, revision bariatric surgery, or emergency surgery

© 2020 Sun Y et al. *JAMA Network Open.*

[b]The association between pre-operative BMI reduction percentage and intra-operative or 30-day post-operative mortality among 425864 participants excluding patients who had previous bariatric surgery, revision bariatric surgery, or emergency surgery
[c]The association between pre-operative weight loss percentage and intra-operative or 30-day post-operative mortality among 305332 participants undergoing Sleeve
[d] Adjusted for covariates included in model 1 plus a history of previous bariatric surgery, if the bariatric surgery was a revision or conversion, and if the patient underwent emergency surgery during the hospital admission.
[e]The association between pre-operative weight loss percentage and intra-operative or 30-day post-operative mortality among 126362 participants undergoing RYGB
[f]Adjusted for covariates included in model 1 plus a history of previous bariatric surgery, if the bariatric surgery was a revision or conversion, and if the patient underwent emergency surgery during the hospital admission.

© 2020 Sun Y et al. *JAMA Network Open.*
